# Assessment of inflammatory suppression and fibroblast infiltration in tissue remodelling by supercritical CO_2_ acellular dermal matrix (scADM) utilizing Sprague Dawley models

**DOI:** 10.3389/fbioe.2024.1407797

**Published:** 2024-06-24

**Authors:** Nguyen Ngan Giang, Linh Thi Thuy Le, Pham Ngoc Chien, Thuy-Tien Thi Trinh, Pham Thi Nga, Xin Rui Zhang, Yong Xun Jin, Shu Yi Zhou, Jeonghun Han, Sun Young Nam, Chan Yeong Heo

**Affiliations:** ^1^ Department of Medical Device Development, College of Medicine, Seoul National University, Seoul, Republic of Korea; ^2^ Department of Plastic and Reconstructive Surgery, Seoul National University Bundang Hospital, Seongnam, Republic of Korea; ^3^ Department of Biomedical Science, College of Medicine, Seoul National University, Seoul, Republic of Korea; ^4^ Faculty of Medical Technology, Haiphong University of Medicine and Pharmacy, Haiphong, Vietnam; ^5^ Korean Institute of Nonclinical Study, H&Bio Co., Ltd., Seongnam, Republic of Korea; ^6^ Department of Medicine, College of Medicine, Seoul National University, Seoul, Republic of Korea; ^7^ DOF Inc., Hwaseong, Republic of Korea

**Keywords:** ADM, implant, breast surgery, cell proliferation, capsule formation

## Abstract

Human skin-derived ECM aids cell functions but can trigger immune reactions; therefore it is addressed through decellularization. Acellular dermal matrices (ADMs), known for their regenerative properties, are used in tissue and organ regeneration. ADMs now play a key role in plastic and reconstructive surgery, enhancing aesthetics and reducing capsular contracture risk. Innovative decellularization with supercritical carbon dioxide preserves ECM quality for clinical use. The study investigated the cytotoxicity, biocompatibility, and anti-inflammatory properties of supercritical CO_2_ acellular dermal matrix (scADM) *in vivo* based on Sprague Dawley rat models. Initial experiments *in vitro* with fibroblast cells confirmed the non-toxic nature of scADM and demonstrated cell infiltration into scADMs after incubation. Subsequent tests *in vitro* revealed the ability of scADM to suppress inflammation induced by lipopolysaccharides (LPS) presenting by the reduction of pro-inflammatory cytokines TNF-α, IL-6, IL-1β, and MCP-1. In the *in vivo* model, histological assessment of implanted scADMs in 6 months revealed a decrease in inflammatory cells, confirmed further by the biomarkers of inflammation in immunofluorescence staining. Besides, an increase in fibroblast infiltration and collagen formation was observed in histological staining, which was supported by various biomarkers of fibroblasts. Moreover, the study demonstrated vascularization and macrophage polarization, depicting increased endothelial cell formation. Alteration of matrix metalloproteinases (MMPs) was analyzed by RT-PCR, indicating the reduction of MMP2, MMP3, and MMP9 levels over time. Simultaneously, an increase in collagen deposition of collagen I and collagen III was observed, verified in immunofluorescent staining, RT-PCR, and western blotting. Overall, the findings suggested that scADMs offer significant benefits in improving outcomes in implant-based procedures as well as soft tissue substitution.

## 1 Introduction

Allotransplantation, or allografting, is gaining global attention for its efficacy in cultivating fully formed skin and transplants of damaged organs (Carrier et al., 2022; Gotlieb et al., 2022; Cheng et al., 2023). The demonstrated potential in plastic and reconstructive surgery highlights the need for continuous improvement to meet modern demands in which products must be advanced and become more biocompatible for clinical applications (Al-Himdani et al., 2017; Peng et al., 2020).

The extracellular matrix (ECM), derived from human skin, serves as a reservoir of growth factors influencing biodynamics, such as cell proliferation, migration, differentiation, and apoptosis (Yue, 2014; Huang et al., 2022; Trinh et al., 2022). It is comprised of fibrins, fibronectin, collagens, and hyaluronic acid, accelerating dermal formation and serving as an ideal scaffold for tissue bioengineering. However, due to being composed of several cell types, natural ECMs may trigger immunologic reactions, leading to inflammation (Pfisterer et al., 2021; Kasravi et al., 2023). To address this, decellularization processes have been initiated to prevent adverse reactions caused by cellular components while maintaining the ECM’s native structures by eliminating immunogenic cellular components, which are often subjected to terminal sterilization to minimize bioburden (Barbulescu et al., 2022; Zhang et al., 2022).

A popular product among decellularized ECMs (dECMs) is the acellular dermal matrix (ADM), recommended for augmentation as bioscaffolds that emphasize native ECMs and support functional regeneration of organs after transplantation (Petrie et al., 2022; Xiao et al., 2023; Zhang et al., 2023). The reports of ADM fabrication were published in which traditional decellularization methods using harsh polar detergents may denature ECM proteins due to solvent properties (Mendibil et al., 2020; Xiao et al., 2023). An appealing alternative by DOF company involves using a supercritical fluid of carbon dioxide as a solvent for decellularization at low temperatures, preventing denaturation and enhancing permeability into micropores, particularly for DNA extraction was discussed in our previous study (Trinh et al., 2022).

On the basis of their regenerative properties, ADMs were initially utilized in 1992 as a dermal substitute for severe burn treatment, then subsequently continued to be applied as soft-tissue replacements until now (Buinewicz and Rosen, 2004). The growing availability of ADMs offers plastic surgeons a valuable tool to enhance the aesthetic outcomes of implant-based reconstruction by expanding and reshaping the implant pocket, while also reducing the invasiveness of surgery (Gamboa-Bobadilla, 2006; Scheflan and Colwell, 2014). As well, ADMs facilitate breast contouring, reduce the risk of capsular contracture post-implant insertion, and reliably sustain the positioning of the reconstructed breast (Gravina et al., 2019). Over time, ADMs have been increasing their uses in breast surgery, particularly proving beneficial in the placement of tissue expanders and implants during primary breast reconstruction (Spear et al., 2008).

In this study, we implanted a decellularized ADM-derived human skin allografting product by applying supercritical carbon dioxide fluid (scADM) *in vivo* to imitate the breast implantation operation. This work aimed to provide more reliable evidence on whether scADM can provide tissue formation through cell infiltration into the implant by characterizing cell proliferation factors such as Vimentin, SMA, and Ki67. A general histological assessment examination and a Western blot were performed to investigate whether the internal collagen matrix formation is presented inside the scADM after implantation by investigating collagen genesis (Collagen I) and degradation (MMP family). Furthermore, the study also evaluated capsular contracture formation, which may help provide a specific reference value for plastic surgeons.

## 2 Materials and reagents

The scADM were from DOF company (Seoul, South Korea). The product was created according to the instructions in a pending patent.

The methods of ADM preparation were reported in the previous study (Giang et al., 2022). In brief, the tissue was defatted, washed in sterile phosphate-buffered saline (PBS) for 30 min, and treated with 1 M NaCl at 37°C overnight to separate the epidermis from the dermis. Supercritical carbon dioxide (scCO2) extraction was performed using homemade equipment, with the skin tissue soaked in PBS with 1% antibiotics on a 30 rpm rocker overnight. Ethanol (30%–70%) was added as a co-solvent, and the extraction was conducted at 200–400 bar for about 3 h. Post-extraction, the ADM was washed in PBS overnight, cut into 10(L) × 10(W) × 2(H) mm pieces, treated with maltitol for lyophilization, and sterilized with 15 kGy gamma irradiation.

### 2.1 MTT assay

The MTT assay method was used to examine the vitality of cells in two groups: non-treated (the control group) and cells treated with scADM (the scADM group) after 24, 48, and 72 h scADM samples, cut into 1 cm^2^ pieces, were immersed in a Phosphate buffered saline (PBS) (Gibco, Thermo Fisher Scientific, MA, United States) containing penicillin-streptomycin (PS) for 24 h, followed by incubation in Dulbecco’s Modified Essential Medium High Glucose, with L-Glutamine (DMEM) (Gibco, Thermo Fisher Scientific, MA, United States) culture medium with 10% Fetal Bovine Serum (FBS) (Gibco, Thermo Fisher Scientific, MA, United States) and 1% PS for an additional 24 h. Mouse fibroblast cell lines, NIH-3T3, (Korean Cell Line Bank) were seeded in 24-well plates at 4 × 10^4^ cells/well. The cells in the control group were cultured in complete medium, whereas the cells in the scADM group were seeded and subsequently exposed to scADM positioned above them in the culture setup, maintained at 37°C, 5% CO_2_. After the treatment period, 200 µL of 3-(4,5-dimethylthiazol-2-yl)-2,5-diphenyltetrazolium bromide (MTT) solution was added to each well and incubated for 4 h in the incubator. Then, the media was removed, and the plate was rinsed with PBS. The formazan product was dissolved in Dimethyl sulfoxide (DMSO) (Chemicals Duksan, Gyeonggi-do, Korea) and the absorbance intensity was measured at a wavelength of 570 nm.

### 2.2 Cell infiltration

scADM were immersed in PBS buffer with 1% PS for 24 h, followed by a 24-h incubation in DMEM culture medium supplemented with 10% FBS and 1% PS at 37°C, 5% CO_2_ for 24 h. The scADM tissues were cut uniformly 1 cm^2^ and placed into a 24-well plate. Subsequently, the mouse fibroblast cell line NIH-3T3 (4 × 10^4^ cells/well) was then added to the surfaces of the scADM and proliferated for up to 14 days. Following this, the tissues were fixed in 10% formalin for 24 h at 4°C and then embedded in paraffin blocks in preparation for Hematoxylin and Eosin (H&E) staining of tissue sections. The infiltration of cells into the ADM was verified through microscopic imaging.

### 2.3 ELISA

Raw 264.7 cells were plated at 2 × 10^5^ cells/well in 24 well-plate and cultured at 37°C, 5% CO_2_ for 24 h. Subsequently, the cells were divided into four treatment groups: the control group, the LPS group (cells treated with 1 μg/mL of Lipopolysaccharides), the scADM group (cells cultured under scADM), and the LPS + ADM group (cells cultured under scADM and treated with 1 μg/mL of LPS). After 24 h of treatment, the supernatant from each group was collected for ELISA analysis.

For Elisa analysis, a 96-well plate was coated overnight at 4°C with capture antibodies against TNF-α, IL-6, MCP-1, and IL-1β from BD Biosciences (NJ, United States), diluted 1:500 in PBS. After coating, the plate was washed and blocked with a blocking buffer containing 10% FBS in PBS to prevent non-specific binding. Standards and supernatant samples were diluted with the blocking buffer, added to the wells, and incubated for 3 h at 25°C. A second antibody (1:500 in blocking buffer, BD Biosciences, NJ, United States) was added for a further 2-h incubation for 2 h incubation. Following another wash, avidin-peroxidase (Sigma-Aldrich, MO, United States) was applied for 30 min, Subsequently, 100 µL of a 1:1 solution of substrate reagents A and B (BD OptEIA TMB Substrate Reagent Set, BD Biosciences, NJ, United States) was added to each well. The absorbance was measured at 405 nm using a BioTek Epoch 2 microplate spectrophotometer (BioTek Instruments, VT, United States) for quantitative analysis of the specific proteins. The standard curve was prepared using 12 points with a 2-fold dilution starting from 10 ng/mL of recombinant Rat TNF, IL-6, MCP-1, and IL-1β proteins from BD Biosciences (NJ, United States).

### 2.4 Determination of nitrite production in cell supernatant

Nitrite and NO oxidation products present in the cell supernatant were analyzed using the Griess reagent modified (Sigma-Aldrich, MO, United States). Specifically, 50 µL of the supernatant was combined with 50 µL of 1× Griess reagent (modified), and incubated at room temperature for 15 min. The 1× Griess reagent (modified) was prepared according to the manufacturer’s instruction by mixing 10 g of Griess reagent (modified) in 250 mL of water, followed by inverting the bottle for about 5 minutes. To establish a standard curve for quantification, a series of nitrite standard solutions was prepared with a 2-fold dilution, ranging from 0 to 50 µM. The absorbance was measured using a microplate spectrophotometer BioTek Epoch 2 (BioTek Instruments, VT, United States) at a wavelength of 504 nm.

### 2.5 *In vivo* experiment: ADM implantation in rat

Animal experiments were conducted following approval by the Institutional Animal Care and Use Committee of Seoul National University Bundang Hospital (approval number: BA-2111-332-006-18). Eight-week-old Sprague Dawley adult male rats (BioOrient Company, Seongnam, Korea) weighing between 200 and 350 g were housed in pairs with access to food and water *ad libitum*. The rats were housed in a controlled environment maintained under specific-pathogen-free (SPF) conditions, with a 12-h light/dark cycle, relative humidity of 55%, and a temperature of 24°C.

On the day of surgery, twenty rats were divided into 4 groups at random: 1-month scADM (*n* = 5), 2-month scADM (*n* = 5), 3-month scADM (*n* = 5), 6-month scADM (*n* = 5). After being anesthetized with Isoflurane, the dorsal hair along the connecting line between the spine and the anterior superior iliac spine was removed. The subcutaneous graft implantation site was then disinfected, and a 2 cm incision was made along the sagittal position at the center of this area. scADM grafts of uniform size (1 cm^2^) were carefully implanted into the sub-panniculus pocket of each group, and the incision site was sutured after achieving hemostasis. The implanted rats were closely monitored during recovery and provided *ad libitum* access to food and water post-operation. At 1, 2, 3, and 6 months post-surgery, the rats from each group (1-month scADM, 2-month scADM, 3-month scADM, and 6-month scADM) were sacrificed and biopsied, respectively. The biopsy area was tissue near the implanted dorsal region, including the epidermis, dermis, posterior and anterior capsules.

### 2.6 Histological analysis

The biopsied tissues were immersed in a 10% formaldehyde solution for 24 h and then embedded in paraffin. The tissue samples were cut into 3 µm thick slides. These section slides were used for histological examinations in the future.

### 2.7 H& E staining

The tissue sections were deparaffinized in xylene for 3 min two times, followed by rehydration in a different reducing ethanol concentration. After staining with Hematoxylin for 5 min, the sections were rinsed and incubated in Bluing reagent, then washed and stained with Eosin Y for 3 min. Finally, the sections were dehydrated in ethanol, mounted with synthetic resin, and coverslipped. The H&E staining sections were examined using a microscope (AX10, ZEISS, Germany) to observe the cell infiltration into scADM and count number of inflamatory and fibroblast cells.

### 2.8 MT staining

The tissue slides were processed according to the manufacturer’s instructions for Masson’s trichrome staining. After deparaffinization and rehydration, the slides were stained with Bouin overnight at room temperature and rinsed until transparent. Next, they were stained with Weigert’s Hematoxylin for 10 min, followed by immersion in Biebrich scarlet acid solution for another 10 min. The samples were cleaned and differentiated sequentially in phosphomolybdic-phosphotungstic acid and aniline blue solutions for 10 min each. After rinsing with water and incubating in acetic acid for 3 min, dehydration was carried out using 100% ethanol for 3 min, 95% ethanol for 2 min, and 10 min in xylene. The tissue slides were mounted with a mounting medium. Collagen fiber production and intensity were observed and quantified using a microscope and ImageJ software.

### 2.9 Immunofluorescent staining

Tissue sections underwent deparaffinization and hydration, followed by blocking non-specific binding sites using 4% BSA in PBS at room temperature for 1 hour. Subsequently, the sections were incubated overnight at 4°C with primary antibodies against Vimentin, α-SMA Collagen I, Ki-67, F4/80, Arg-1, CD31, and IL-6 (1:200, Santa Cruz Biotechnology, TX, United States). After washing, the tissue sections were incubated with the secondary antibody, Alexa Fluor 488 goat anti-mouse (1:1000, Invitrogen, Thermo Fisher Scientific, MA, United States), for 1 h at room temperature in the dark. Nuclear labeling was then performed using 4′,6-diamidino-2-phenylindole (Fluoroshield with DAPI, Sigma-Aldrich, MO, United States) for fluorescence imaging, and the samples were stored at 4°C. Fluorescent signals were captured using a confocal microscope Zeiss LSM 710 (ZEISS group, Oberkochen, Germany). The images were analyzed by ImageJ software (The National Institutes of Health, Bethesda, MD, United States) to measure the intensity of the biomarkers, including inflammatory cytokines and proliferation markers. This analysis facilitated the quantification and comparison of biomarker expression levels in the tissue sections.

### 2.10 Western blot analysis

The Western blot experiment was conducted to analyze the expression of collagen I, MMP-9, and beta-actin from implantation tissue collected in an *in vivo* experiment. Tissues from the four groups, collected at 1, 2, 3, and 6 months post-surgery, were snap-frozen in liquid nitrogen. To extract protein, the frozen tissues were homogenized in RIPA Lysis Buffer (Merck Millipore, Darmstadt, Germany, Ready-to-use solution containing 150 mM NaCl, 1.0% IGEPAL^®^ CA-630, 0.5% sodium deoxycholate, 0.1% SDS, 50 mM Tris, pH 8.0) containing Protease Inhibitor Cocktails (Roche, Basel, Switzerland) The homogenate was centrifuged at 14,000 ×g for 15 min at 4°C to collect the supernatants. Protein concentrations were determined using the Pierce BCA Protein Assay Kit (Thermo Fisher Scientific, MA, United States). Proteins were separated by sodium dodecyl sulfate-polyacrylamide gel electrophoresis using the Mini-PROTEAN Precast gels system (Bio-Rad Laboratories, CA, United States) and transferred onto nitrocellulose membranes using the Trans-Blot Turbo system (Bio-Rad Laboratories, CA, United States). After blocking with 5% nonfat skim milk in TBS-T (Tris-buffered saline with 0.1% Tween), membranes were incubated overnight at 4°C with primary antibodies: mouse anti-beta-actin (1:1000, Santa Cruz Biotechnology, TX, United States), anti-collagen I (1:500, Santa Cruz Biotechnology, TX, United States), and anti-MMP-9 (1:1000, Santa Cruz Biotechnology, TX, United States). Membranes were then incubated with a secondary antibody, goat anti-mouse IgG (H + L)-HRP conjugate (1:2000, Bio-Rad Laboratories, CA, United States), for 2 h. Protein expression levels were visualized using WestGlow TM PICO PLUS chemiluminescent substrate (Biomax, Seoul, Korea) and imaged with a ChemiDoc™ Imaging System (Bio-Rad Laboratories, CA, United States). The expression of target proteins was quantified using ImageJ software and normalized to beta-actin expression, enabling accurate assessment and comparison of protein expression levels across samples and time points.

### 2.11 Real time-PCR

After 1, 2, 3, and 6 months post-surgery, the skin tissue was collected, and total RNA was extracted using RNAiso Plus (Takara Bio, Shiga, Japan) according to the manufacturer’s instructions. Subsequently, cDNA synthesis was performed from approximately 1 µg of total RNA according to the protocol provided by the RevertAid First Strand cDNA Synthesis Kit (Thermo Fisher Scientific, MA, United States). Specific primer pairs analyzed the expression of MMP-2, MMP-3, MMP-9, Collagen I, and GAPDH were follows: MMP-2 forward 5′- CAC AGA CCC TGC TCT AAT GC -3′, MMP-2 reverse 5′- AGG TGT CAG GCT ATG GCA AG -3′, MMP-3 forward 5′- CCT CTG AGT CTT TTC ATG GAG GG -3′, MMP-3 reverse 5′- ACT TGA GGT TGA CTG GTG CC - 3′, MMP-9 forward 5′- GAT CCC CAG AGC GTT ACT CG -3′, MMP-9 reverse 5′- GTT GTG GAA ACT CAC ACG CC -3, Collagen I forward 5′- CCG TGA CCT CAA GAT GTG C -3′, Collagen I reverse 5′- GAA CCT TCG CTT CCA TAC TCG-3′, GAPDH forward 5′- CGC TAA CAT CAA ATG GGG TG -3′, GAPDH reverse 5′- TTG CTG ACA ATC TTG AGG GAG-3'. For quantitative PCR, TB Green^®^ Premix Ex Taq™ II (Takara Bio, Shiga, Japan) was used on the QuantStudio™ 3 Realtime PCR (Thermo Fisher Scientific, MA, United States) with the following PCR conditions: initial denaturation at 95°C for 30 s, followed by 45 cycles of amplification at 95°C for 5 s and 60°C for 34 s. Relative mRNA expression values were determined using the comparative CT method, the 2^−ΔΔCT^ method.

### 2.12 Statistical analysis

The data were displayed as mean value ± standard of the mean bar from repeating three to five independent experiments using GraphPad Prism software (GraphPad Software Inc, SanDiego, CA, United States), and statistical analysis was performed by comparison between groups. A *p-value* less than 0.05 was considered statistically significant in the unpaired *t*-test.

## 3 Results

### 3.1 Cell infiltration evaluation of acellular dermal matrix (ADM) *in vitro*

**FIGURE 1:**
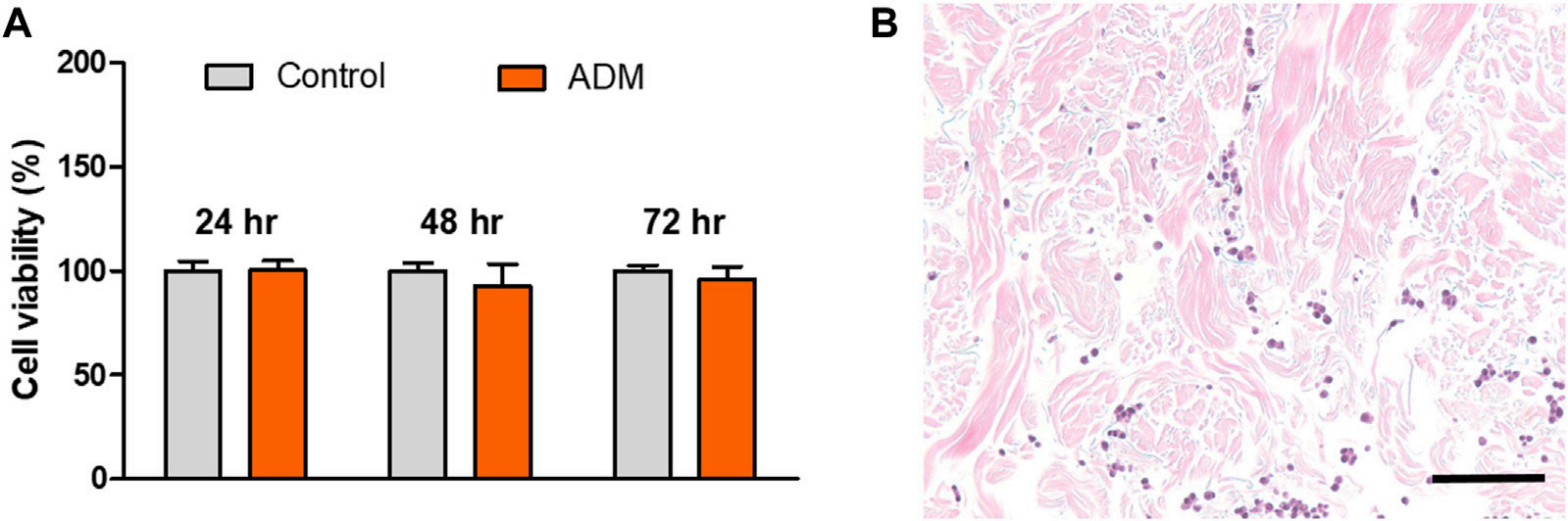
**(A)** Cell viability by MTT assay and **(B)** Cell infiltration into the acellular dermal matrix (ADM) *in vitro*.

The cytotoxicity of the scADM to fibroblast cells was performed in 24, 48, and 72 h by using an MTT assay, in which the viability rate was ascribed to 100% as the maxima for cell viability. As shown in Figure 1A, the rate of viability exhibited a trivial change in OD intensity between the control groups and the ADM groups, verifying the non-toxicity of the ADMs. Cell infiltration experiments were conducted to further confirm the biocompatibility of the ADMs *in vitro*, as shown in Figure 1B. Cell penetration into ADMs was observed after 14 days of fibroblast incubation with ADMs.

### 3.2 Evaluation of inflammatory responses of cells incubated ADMs

**FIGURE 2:**
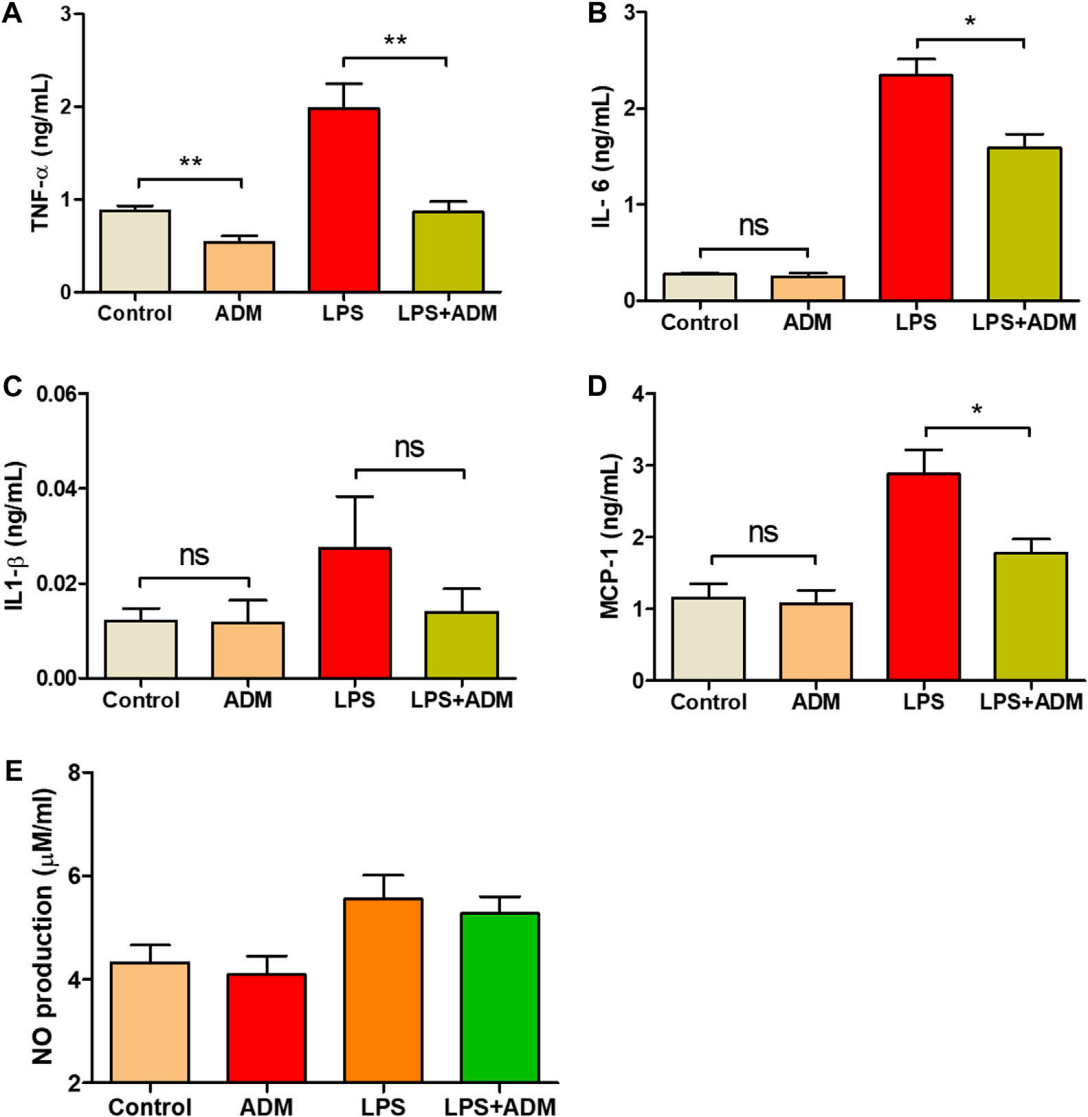
Inflammatory response reduction by ADMs *in vitro* in which **(A)** Tumor necrosis factor alpha (TNF-α) **(B)** Interleukin-6 (IL-6) **(C)** Interleukin-1 beta (IL-1β) **(D)** Monocyte chemoattractant protein 1 (MCP-1) by ELISA and **(E)** Nitric oxide (NO) production by nitrate assay The presented values are the experimental results from *in vitro* experiments. Data are shown as the mean ± SEM. *p ≤ 0.05, **p ≤ 0.005, ***p ≤ 0.001.

To additionally investigate whether the scADM possessed the capability of inflammatory suppression, we leveraged lipopolysaccharides (LPS) as a pro-inflammatory reagent to induce an inflammatory reaction *in vitro*. The concentration of four inflammatory cytokines was measured after the experiment. Figures 2A–D depicted a significant increase in LPS level that proved effective inflammatory activation in cells. Moreover, a significant decrement of TNF- α and IL—6 in the LPS-induced cells with ADMs groups was observed (Figures 2A,B). Similarly, the results of IL-1β and MCP-1 measurements presented an observable reduction in inflammatory secretion when LPS-induced cells coevally incubated with scADM compared with the LPS treatment groups in Figures 2C,D, As shown in Figure 2E, the NO generation level in both control the scADM-cell incubated group exhibited a non-significant difference with 4.33 ± 0.33 uM/mL for control and 4.09 ± 0.36 while the generated NO level in LPS-induced group increased to 5.55 ± 0.46 uM/mL. However, noticeably, there was a slight decrease observed in the LPS-induced scADM group at 5.20 ± 0.33 uM/mL.

### 3.3 Histological assessment for cell infiltration into acellular dermal matrix (scADM) *in vivo*

**FIGURE 3:**
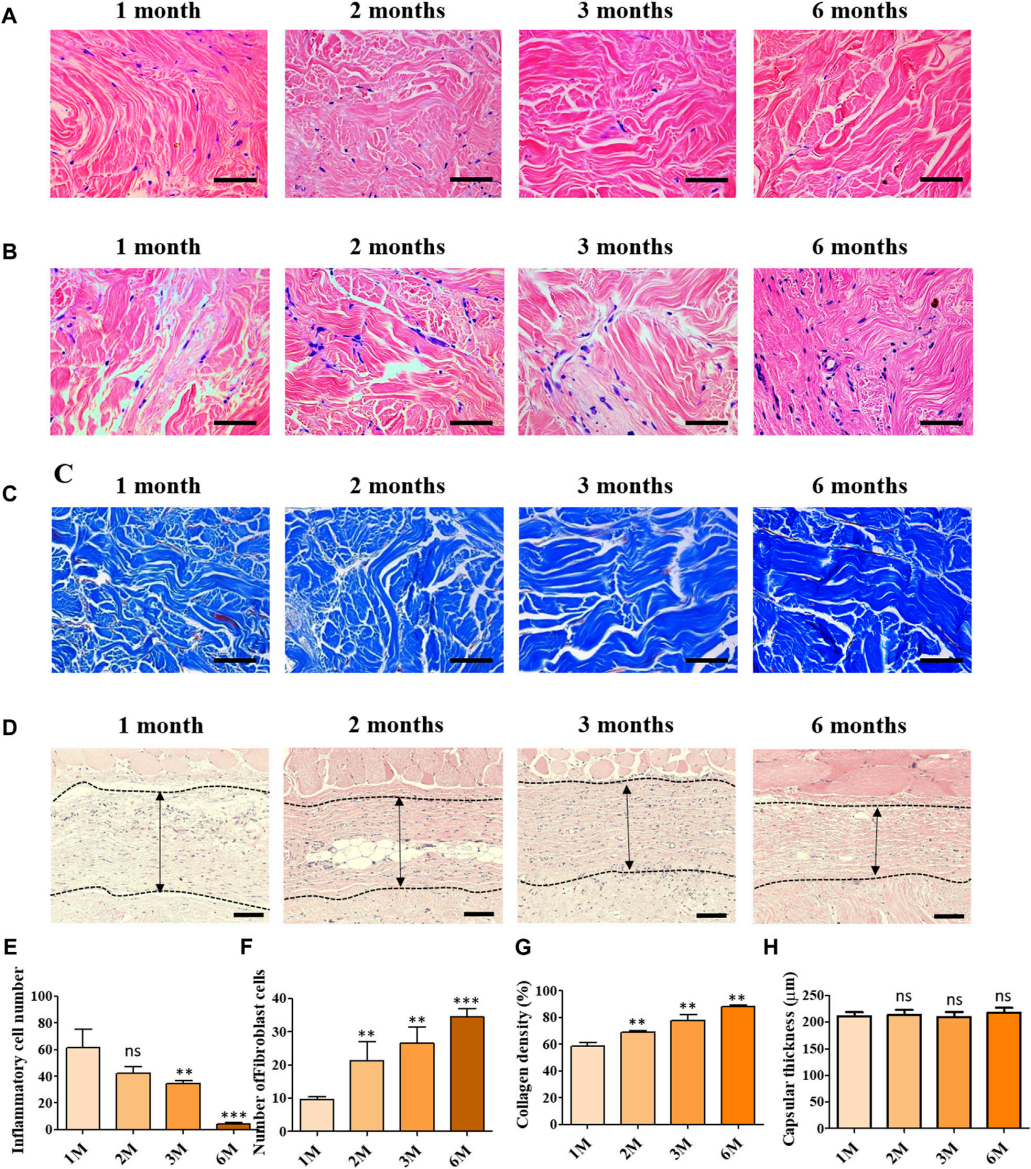
Histological assessment of implanted ADMs *in vivo* in which **(A)** Hematoxylin and eosin **(H and E)** staining for inflammatory cells **(B)** Hematoxylin and eosin **(H and E)** staining of fibroblast cell infiltration **(C)** Masson’s trichrome (MT) staining **(D)** Hematoxylin and eosin **(H and E)** staining for capsular formation **(E)** Quantitative number of inflammatory cells **(F)** Quantitative number of fibroblast cells and **(G)** Quantitative result of collagen density **(H)** Quantitative results of capsular formation. Scale bar = 50 µm. The presented values are the experimental results on 5 rats for each group. Data are shown as the mean ± SEM. **p ≤ 0.005, ***p ≤ 0.001.

Figure 3 depicts the histological images of scADM implanted in rats after 1 month, 2 months, 3 months, and 6 months.

To evaluate the capability of inflammatory suppression of scADM, the inflammatory cells were first identified by H&E staining in sectioned scADMs, as shown in Figures 3A,E. The observed data displayed a gradual reduction in inflammatory cells in the implanted scADMs from 1 month to 6 months, which proves the inhibition of inflammatory cell expansion of the scADM.

According to the H&E staining for inflammatory cells, the sectioned tissues were monitored to evaluate the infiltration of fibroblast cells into the scADM from 1 month to 6 months. In accordance with the observation, Figures 3B,F represented a progressive expansion in fibroblast cell density in the matrix after 6 months, reflecting proliferative activity supported by the scADM.

In addition to that, the collagen formation was analyzed by MT staining, demonstrating a remarkable content of newly formed collagen in the scADM after 6 months of implantation compared to that of 1 month (Figures 3C,G). Data can be monitored by the intense and evenly distributed presence of blue stains appearing on the sectioned tissues.

Capsular formation in implantation was also achieved by H&E staining in which the data exhibited a nonsignificant change in capuslar thichkness after implnatation as shown in Figures 3D,H.

### 3.4 Evaluation of cell proliferation in acellular dermal matrix after implantation

**FIGURE 4:**
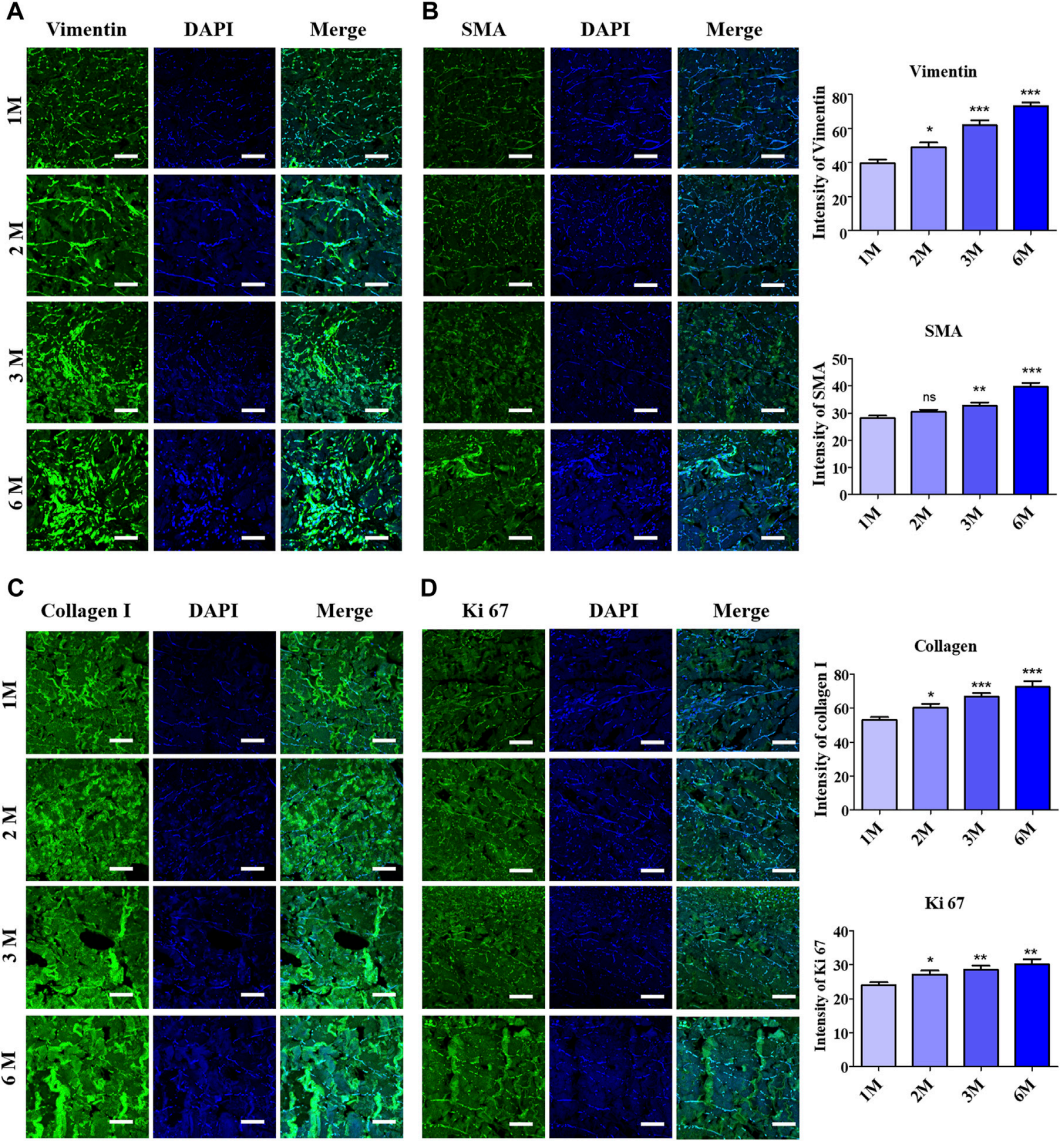
Evaluation of fibroblast infiltration into ADMs after 6 months of implantation by immunofluorescence staining (IF) and quantitative analysis with **(A)** Vimentin, **(B)** Alpha smooth muscle actin (α-SMA) **(C)** Collagen I, **(D)** Ki67. Scale bar = 50 µm. The presented values are the experimental results on 5 rats for each group. Data are shown as the mean ± SEM. **p ≤ 0.005, ***p ≤ 0.001.

Immunofluorescence staining with various proliferation markers was performed to further clarify the acceleration in cell proliferation or cell invasion into the scADM after 6 months of implantation. The representative images of immunofluorescence staining in Figures 4A,B relatively illustrated vimentin and α-SMA, whose markers depict the presence of fibroblasts that visually signified a noticeable increment in the intensity and number of fibroblast cells in the scADM implanted from 1 month to 6 months. Quantitative analysis of both markers showed a similar trend in inclining the intensity of fibroblast biomarkers, as depicted in the figure.

Further investigation on collagen I formation was performed by immunofluorescence staining to determine the newly formed collagen fibers, as shown in Figure 4C, in which the achieved results perceptually revealed progressively elevated collagen fiber formation following 6 months of implantation compared to 1 month. The quantitative analysis of the staining results illustrated the collagen fiber density in 1 month as 53.15 ± 1.66, while the value increased to 72.62 ± 3.34 after 6 months. As a result, unsurprisingly, Ki67 staining manifested a heightened intensity with quantitative data representing an increase from 24.03 ± 0.84 to 30.17 ± 1.51 followed by 1 month to 6 months of implantation, which reflected the continuous cell proliferation into the implant’s mesh (Figure 4D).

### 3.5 Evaluation of inflammatory factors presented in the acellular dermal matrix after implantation

**FIGURE 5:**
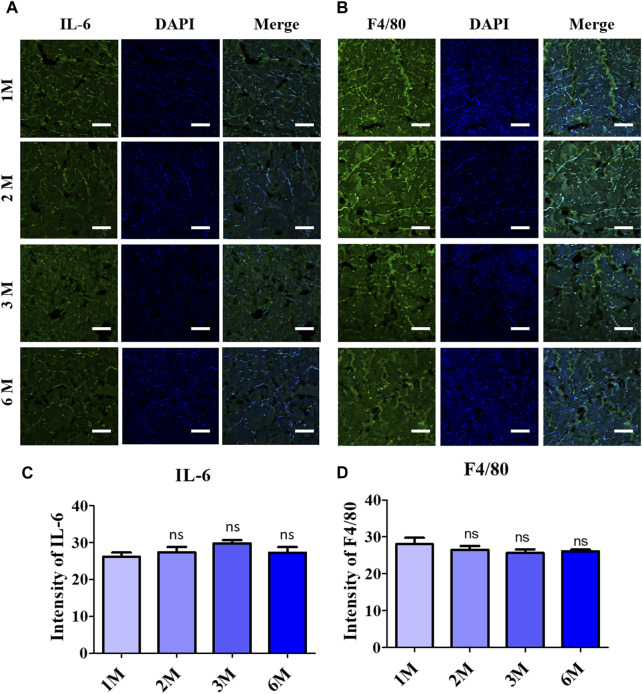
Inflammatory response observation after 6 months of ADMs implantation using immunofluorescence (IF) staining in which **(A)** Interleukin 6 (IL-6), **(B)** F4/80 and quantitative results relatively in **(C)** and **(D)**. Scale bar = 50 µm. The presented values are the experimental results on 5 rats for each group. Data are shown as the mean ± SEM. **p ≤ 0.005, ***p ≤ 0.001.

Immunofluorescence staining was performed to elaborate the response of pro-inflammatory factors after 6 months of implantation, as shown in Figures 5A,B. Quantitative analysis of the IF staining resulted in a non-significant increment of secreted pro-inflammatory proteins into the scADM after implantation for 6 months (Figures 5C,D).

### 3.6 Assessment of remodeling and regenerative acceleration in acellular matrix after implantation

**FIGURE 6:**
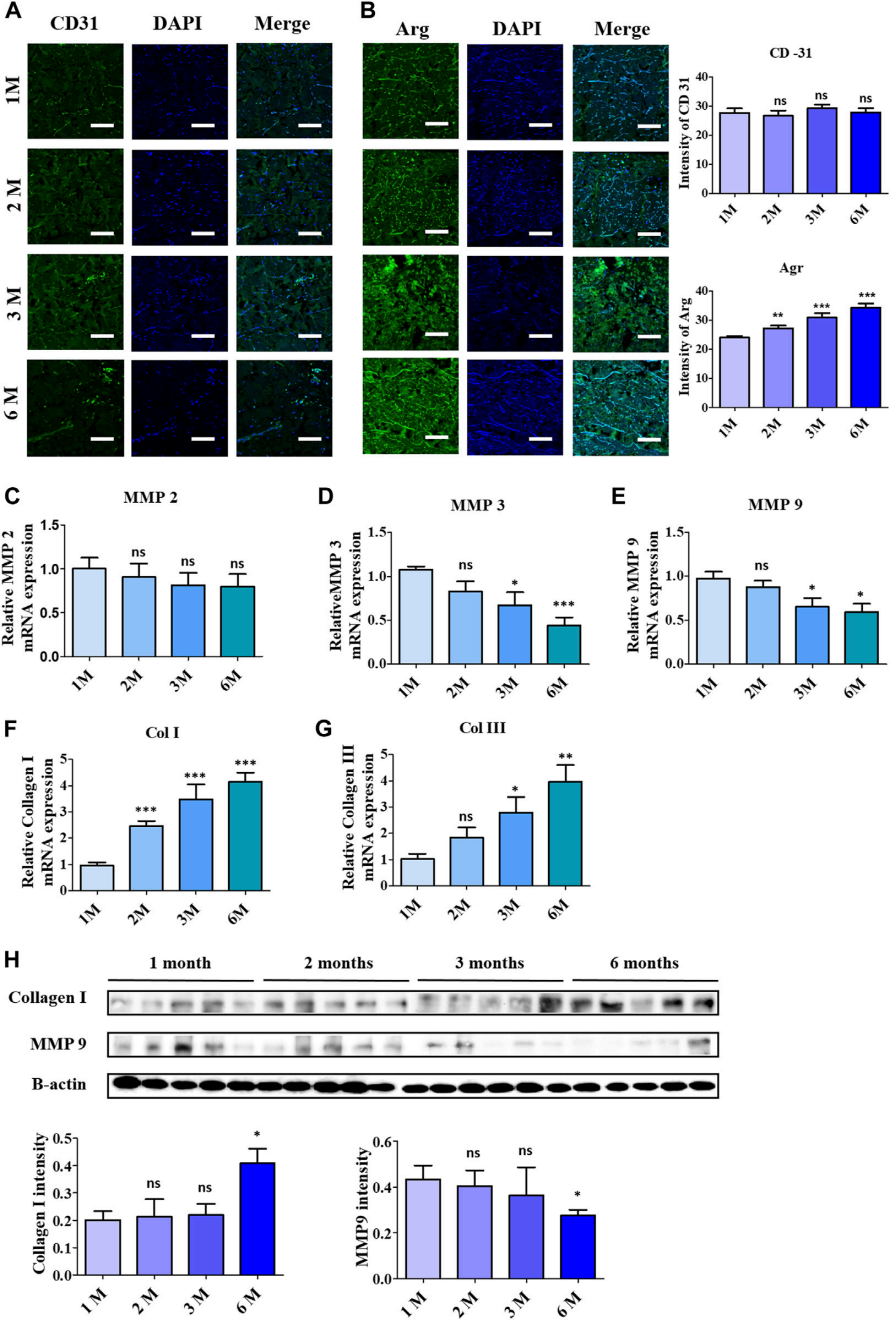
Acceleration of remodelling and vascularization observation after 6 months of ADMs implantation using immunofluorescence (IF) staining and quantitative analysis in which **(A)** CD-31, **(B)** Arginase (Arg 1). Scale bar = 50 µm. The presented values are the experimental results on 5 rats for each group. Data are shown as the mean ± SEM. **p ≤ 0.005, ***p ≤ 0.001. Real-time polymerase chain reaction of remodeling related protein expression in implanted ADMs with **(C)** MMP-2, **(D)** MMP-3, **(E)** MMP-9, **(F)** Collagen 1 and **(G)** Collagen III **(H)** Western blot. The presented values are the experimental results on 5 rats for each group. Data are shown as the mean ± SEM. **p ≤ 0.005, ***p ≤ 0.001.

The regenerative acceleration process was elaborated first by assessing CD31, a vascularization biomarker under immunofluorescence staining, as in Figure 6A, which clearly depicted the formation of endothelial cells at 3 months and 6 months of implantation. Further investigation in the remodeling stage was evaluated by employing Arginase 1, a marker for M2 macrophages representing anti-inflammatory action (Figure 6B). According to that, the visualized images depicted a noticeable increase in fluorescence intensity upon 6-month implantation.

Collagenolysis and elastolysis by matrix metalloproteinases (MMPs) occur in development, wound healing, and inflammatory diseases, including MMP-2, MMP-7, MMP-9, MMP-12, and MT1-MMP. As depicted in Figures 6C–E, MMP groups showed a reduction in mRNA expression after 6 months of implantation, especially MMP3 and MMP9, which complemented the increment of collagen type I and III synthesis, as shown in Figures 6F,G. In addition to the RT-PCR data, the western blotting data in Figure 6H validated the steady collagenases in collagen I by the increase of protein bands, in contrast to the decrease of the protein concentration of MMP9.

## 4 Discussion

Acellular dermal matrix (ADM) application in preventing complications in implant-based breast surgeries or soft tissue substitution has been reported in several studies (Macadam and Lennox, 2012; Gravina et al., 2019; Mohammadyari et al., 2023). ADM is fabricated by the removal of cellular components from the dermal compartment, exhibiting natural bioactive signals, suitable compatibility with living tissues, and mechanical characteristics, which thus functions as a structural scaffold consisting of extracellular matrix components (Nikolova and Chavali, 2019; Dussoyer et al., 2020). Based on its derivation, current ADMs that exist commercially are categorized into human-derived ADMs, porcine-derived ADMs, and bovine-derived ADMs (Almarzouqi et al., 2018; Pires et al., 2022). In this study, we investigated the histological and biochemical characteristics of the novel human-derived ADMs (scADM) produced by DOF company *in vivo*.

The biomechanical characteristics and immunogenic assays between native and decellularized samples (scADM) were characterized in the previous study (Giang et al., 2022). In the case of our product in the ready-to-use state, the elasticity and strength of the original tissue are almost perfectly maintained, while in the case of the freeze-dried state, the elasticity and strength are approximately 1.5 times higher than that of the original tissue, and were equal or better than those of other commercialized products. The histological staining proved an improved and innovative decellularization method by successfully eliminating immunogenic components. Previously, we examined the mechanical characteristics, histological assessment, and remaining ECM components, like collagen, elastin, sGAG, other growth factors, and the scADM tissues’ anti/pro-inflammatory factors after decellularization. Our study demonstrated the retention of growth factors and ECM components, which assist cells in migrating and proliferating, contributing to regeneration potential. The expression of pro-inflammatory factors is represented at a much lower level compared to native and other products, proving the anti-inflammatory ability of the ADMs.

Cellular processes responsible for the development of capsules and capsular contracture have been investigated (Safran et al., 2021; Gorgy et al., 2023). *In vivo*, ADMs act as foreign material in implantation; therefore, they become attracted by fibroblasts, myoblasts, and various immune cells, leading to the capsular formation around the ADMs (Lopez-Mendez et al., 2021; Eslami-Kaliji et al., 2023; Parker et al., 2023). Consequently, to avoid being demolished by capsular contracture, the implant must be biocompatible with the host tissue, promoting positive biological responses without impairing the implant. scADM, in general, have been proven to reduce capsular contracture caused by fibrosis. Karan et al. elaborated that the capsular thickness notably expanded when in direct contact of with the pectoralis muscle, while areas in contact with ADM showed a decrement in capsular thickness (Chopra et al., 2017). In our finding, the thickness of the capsule was measured and calculated by histological assessment, which showed almost no change in the thickness after 6 months of implantation. The data might be resulted from the suppression of inflammation leading to the decreasing inflammatory cells and other immu in implantation ater 6 months. Though there was not a decrease similar to other research, it is promising for scADM material from DOF in the implant industry since the material does not accelerate capsule thickening.

Fibroblasts are the most abundant cell type in charge of producing the ECM protein to scaffold vessels, tissues, muscles, and other structures in place, serve a role in cell development and repair, and are essential for wound healing (Kendall and Feghali-Bostwick, 2014; Plikus et al., 2021). In the *in vivo* model, the migration and proliferation of fibroblast cells through cell invasion into scADM were investigated. The histological assessment visually demonstrated the increase in the number of fibroblast cells in the mesh of scADM, while the data showed a gradual decrease in inflammatory cells following 6-month implantation. Additionally, the immunofluorescent staining for biomarkers of fibroblast cells such as vimentin and SMA depicted a higher intensity in visualizing the expression of those proteins in fibroblast cells, which was supported by the strong cell proliferation evaluated from Ki67 biomarkers.

Collagen is a member of the fibrous protein family dominating in the extracellular matrix (ECM), which provides structural support to the ECM as well as stabilizes growth factors, and regulates cell adhesion and communication between cells and the ECM (Das et al., 2019; Selvaraj et al., 2024). Collagen type I, expressed in nearly all ECM and connective tissues in the human body, is the most abundant type of collagen, with its most significant functional roles in the skin while collagen type III occupies about 8%–11% (Davison-Kotler et al., 2019; Amirrah et al., 2022). Since fibroblasts are widespread mesenchymal cells that produce collagen and other matrix macromolecules to provide structural support for connective tissues, the increase of fibroblast cells is likely to increase collagen production (Freundlich et al., 1986). In this study, the collagen I generated identified by immunofluorescent staining, depicted a noticeable increase in intensity distributed in the mesh implant, following the proliferation of fibroblast cells by time after 6 months of implantation. The data resulted in the new formation of collagen fibers in scADM, which would support the internal matrix of the implant.

Macrophages are considered a good indicator for the biocompatibility evaluation of implants (Lužajić Božinovski et al., 2022; Lu et al., 2023). In which the M1 macrophage generates pro-inflammatory such as IL-6 and TNF- α, while the M2 macrophages function as tissue remodeling and regeneration (Pérez and Rius-Pérez, 2022; Yu et al., 2022). In this study, IF staining was utilized to evaluate the secreted IL-6, resulting in a slight reduction of this pro-inflammatory cytokine, which supported the decrease of the F4/80 macrophage 1 marker after completed implantation (6 months). The data of the macrophage phenotyping verified the anti-inflammatory ability of the scAMD which prevents the implants from destruction. Angiogenesis is essential for physiological processes, especially in ECM remodeling, which is regulated and controlled by several factors, including collagen (Ivanov and Chabbarov, 2023). It is well known that collagen has the function to enhance and assist angiogenesis and vascularization; specifically, collagen I has been demonstrated to promote angiogenesis both *in vitro* and *in vivo* (Twardowski et al., 2007; Nilforoushzadeh et al., 2020; Dasgupta et al., 2023)*.* In contrast to the decrease in F4/80 expression of the M1 phenotype, the staining data of CD31, an angiogenic biomarker, showed a slight increase in neovascularization after 3 months, according to the increase in collagen I intensity reported. Moreover, the heightening of the Arg-1 surface marker of M2 macrophage suggests a regenerative response to scAMD after being implanted for 6 months. The elevation in Arg-1 staining data additionally verified the enhancement in collagen I as a part of remodeling, which was progressively produced into scADMs after 6 months of implantation.

The breakdown of collagen, which plays a crucial role in the development, morphogenesis, tissue remodeling, and repair processes, primarily involves various proteases, with matrix metalloproteinases (MMPs) as the major role (Amar et al., 2017). In contrast to collagenesis, the degradation of collagen fibers by matrix metalloproteinases (MMPs) such as MMP 2, 3, and 9 occurs as part of the development of the extracellular matrix, which modulates immunogenic signaling, accelerates the migration of fibroblasts, keratinocytes, scar formation, and ECM remodeling (Rodríguez-Rodríguez et al., 2018; Cabral-Pacheco et al., 2020). MMP-2, and 9, gelatinase members of the MMP family, exhibit collagenolytic activity, with MMP-2 specifically reported to cleave types I, II, and III collagen (Patterson et al., 2001; Collier et al., 2011). Inflammatory cytokines like TNF-α, IL-6, IL-1β activate MMP-9, produced by neutrophils or macrophages, inducing its secretion within neutrophils, thereby collapsing the extracellular matrix (Khaiboullina et al., 2019; Lee and Kim, 2022). In our study, a noticeable reduction can be observed in the rt-PCR data of MMP2, while there was a significant decrease in gene expression of MMP3 and MMP9 after 6 months. The alleviation of MMP gene expression could be ascribed to the inflammatory suppression ability of the scADM proved in our previous study. Moreover, in this study, the data *in vitro* and vivo shows a stasis in the expression of inflammatory cytokines, implying the downregulated MMPs. The collagen deposition level of collagen I and III measured in RT-PCR revealed an exceptional increase following 1 month to 6 months of implantation. The western blot data also affirmed the reduction of MMP9 protein, which, in contrast, demonstrated the density of collagen I, proving the integrity and longevity of the ADM.

## 5 Conclusion

This study discussed the application of DOF company’s acellular dermal matrix (scADM) in breast surgery and soft tissue substitution. Through histological and immunogenic aspects, it highlighted the scADM’s bioactive ability and biocompatibility in an *in vivo* model. Investigation into fibroblast migration and proliferation within scADMs reveals a rise in fibroblast population and subsequent collagen synthesis. Macrophage phenotyping demonstrates the anti-inflammatory properties of scADM, coupled with enhanced neovascularization post-implantation. Additionally, the study evaluates collagen degradation mediated by matrix metalloproteinases (MMPs), highlighting decreased MMP levels and augmented collagen deposition over time, corroborating the durability and resilience of scADM.

## Data Availability

The original contributions presented in the study are included in the article/Supplementary Material, further inquiries can be directed to the corresponding authors.
