# Supplementary figures and images for: Assessment of inflammatory suppression and fibroblast infiltration in tissue remodelling by supercritical CO2 acellular dermal matrix (scADM) utilizing Sprague Dawley models
Source: Front Bioeng Biotechnol. 2024 Jun 24;12:1407797. doi: 10.3389/fbioe.2024.1407797 (PMC11228881; doi:10.3389/fbioe.2024.1407797)

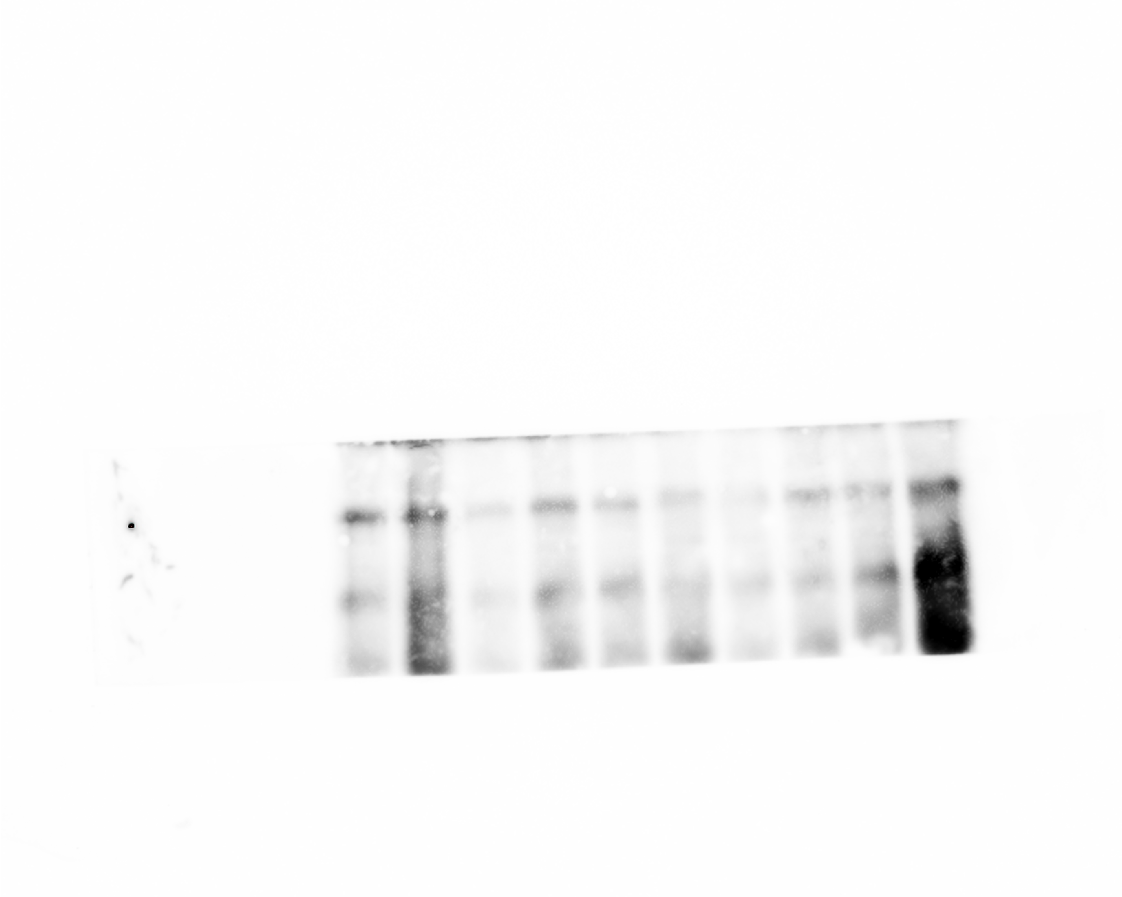

Supplement: Supplementary file 1 [file DataSheet1.ZIP › MMP9 3-6.tif]

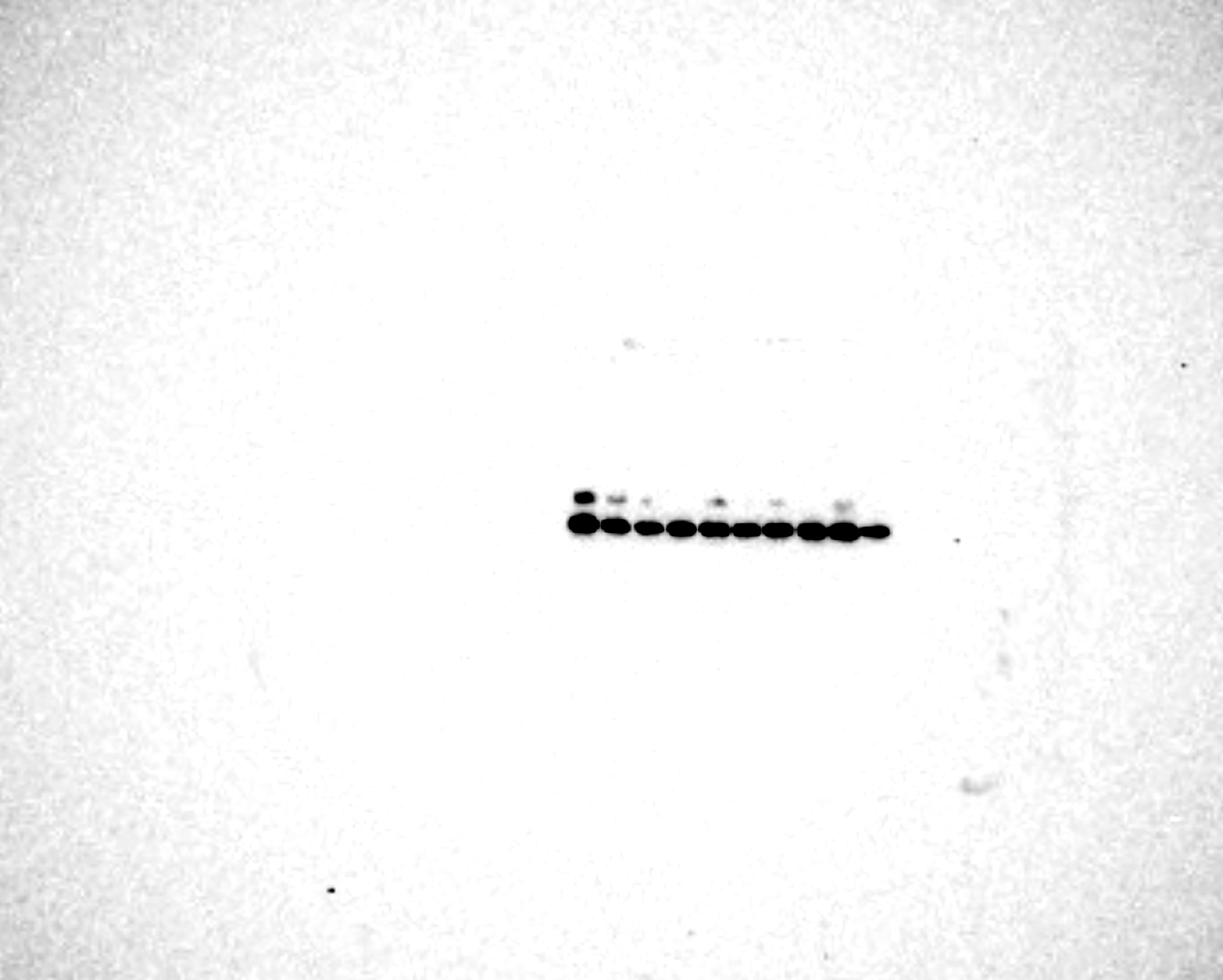

Supplement: Supplementary file 1 [file DataSheet1.ZIP › Beta actin 1-2.tif]

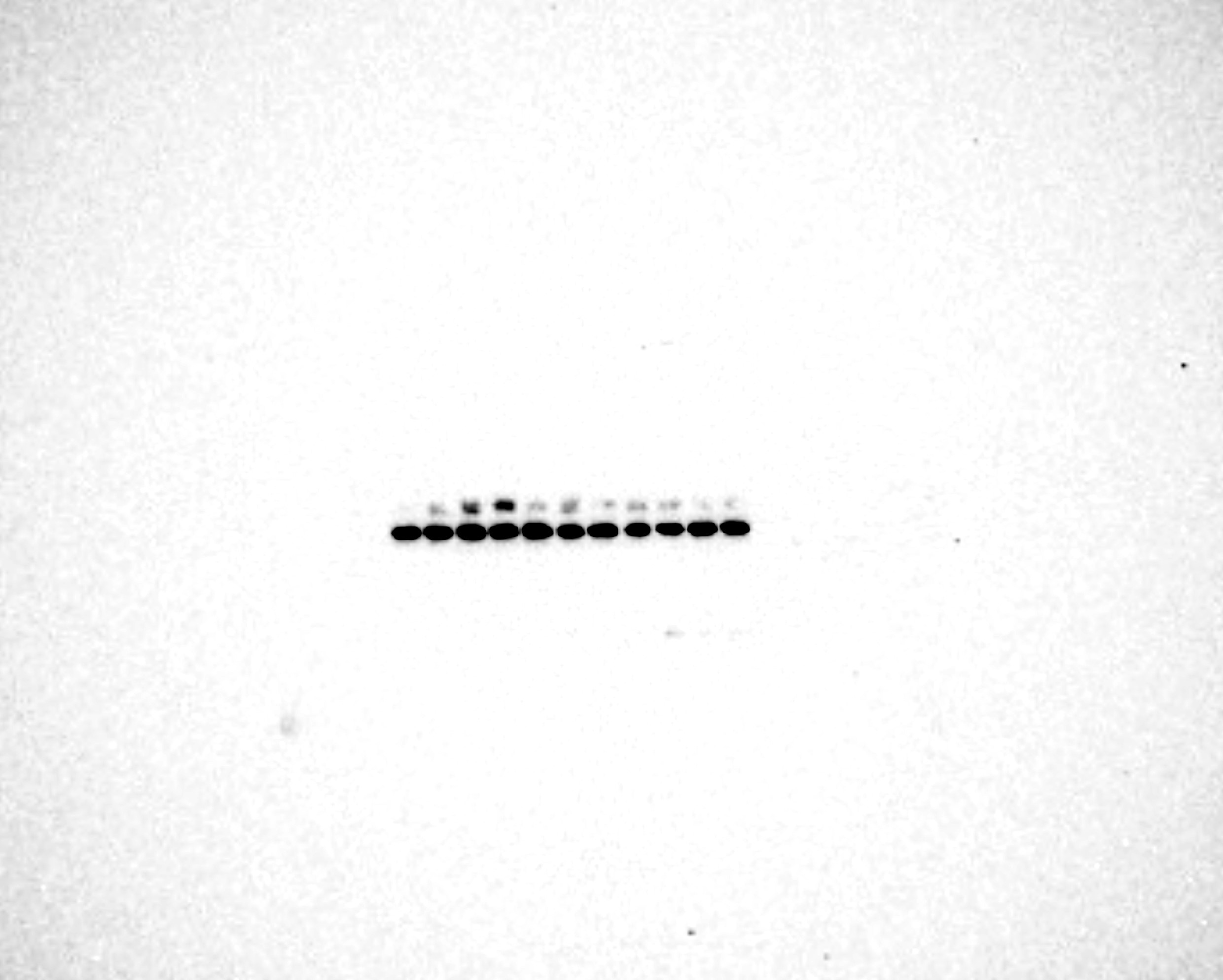

Supplement: Supplementary file 1 [file DataSheet1.ZIP › Beta actin 3-6.tif]

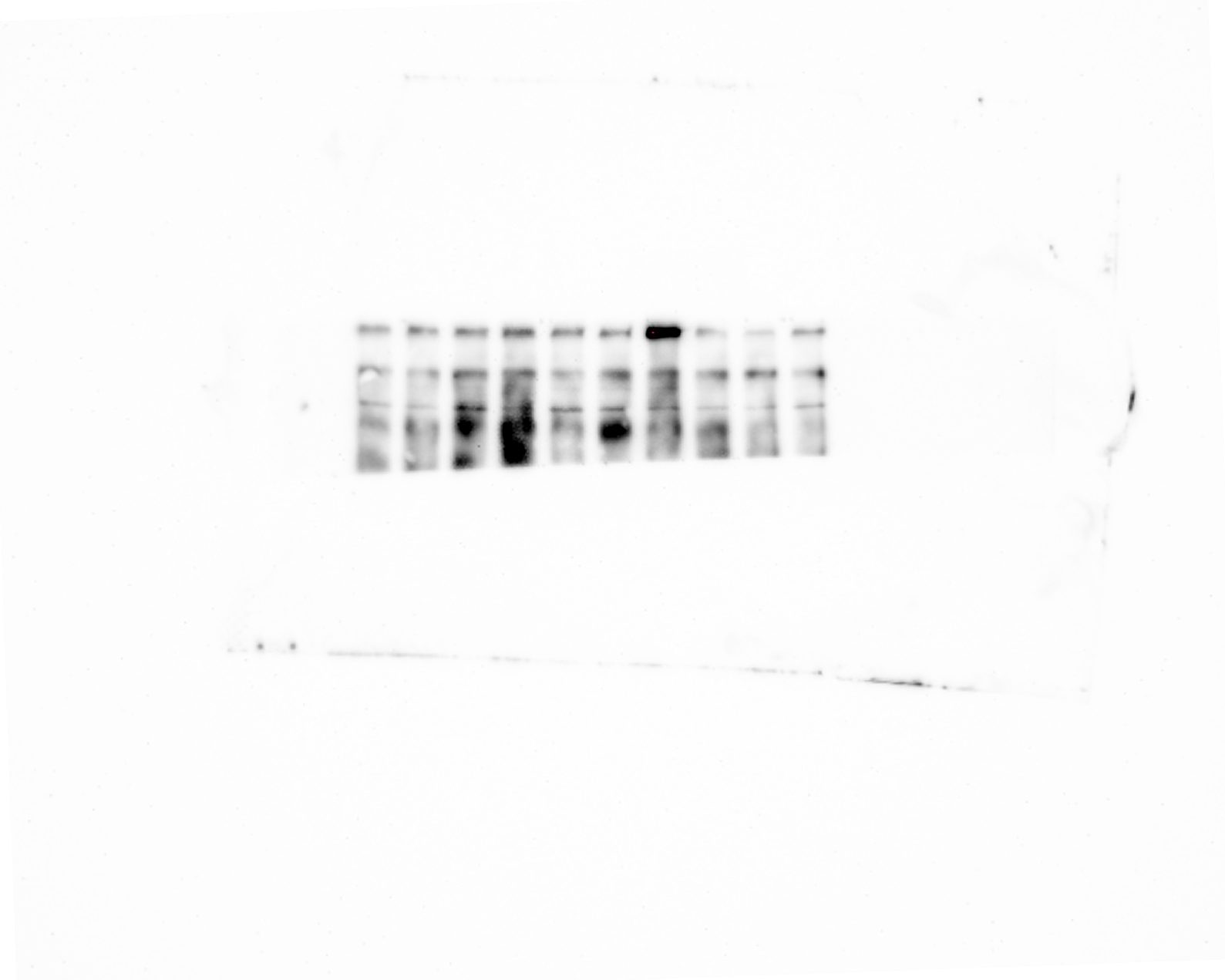

Supplement: Supplementary file 1 [file DataSheet1.ZIP › COL 1_1-2.tif]

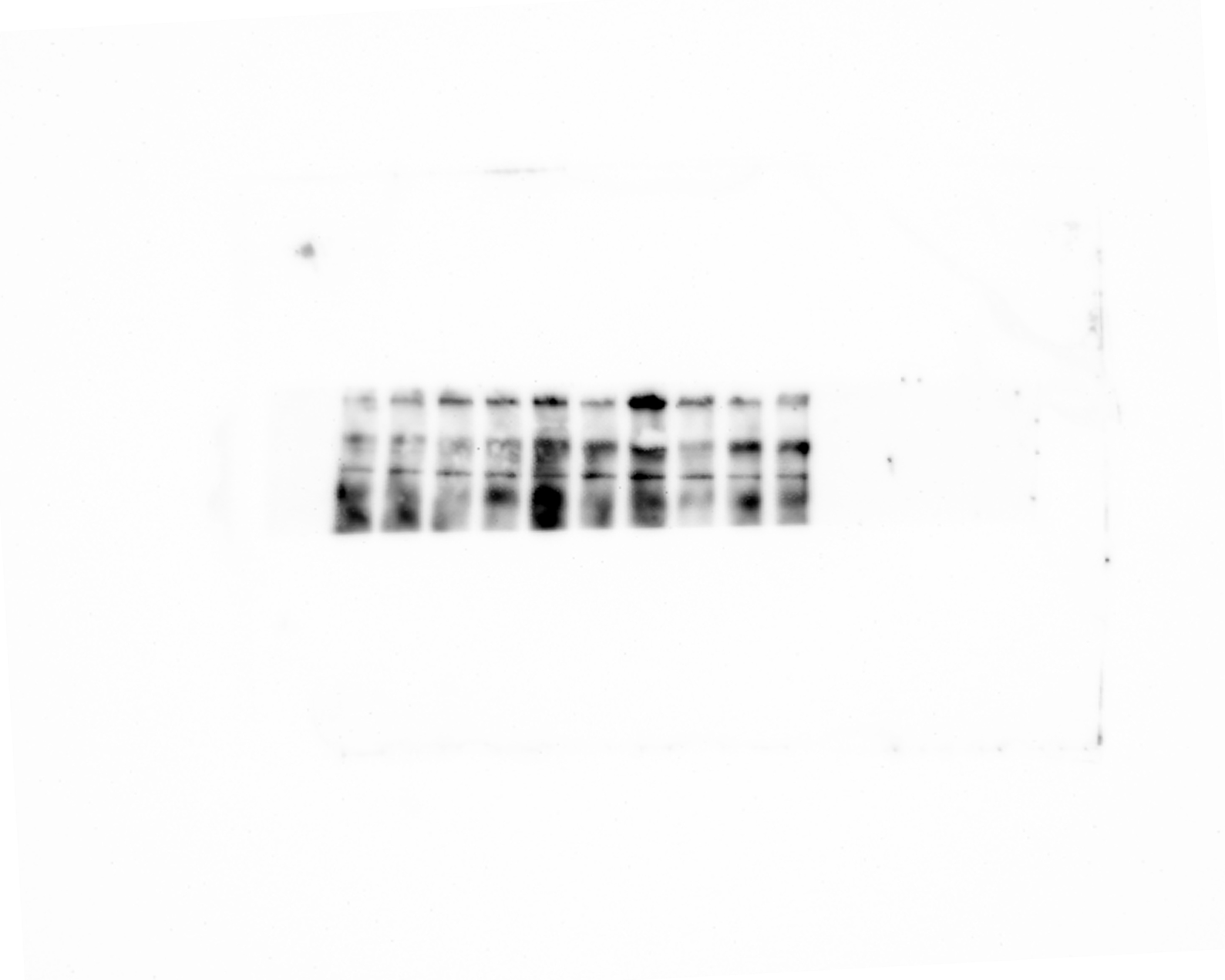

Supplement: Supplementary file 1 [file DataSheet1.ZIP › COL 1_3-6.tif]

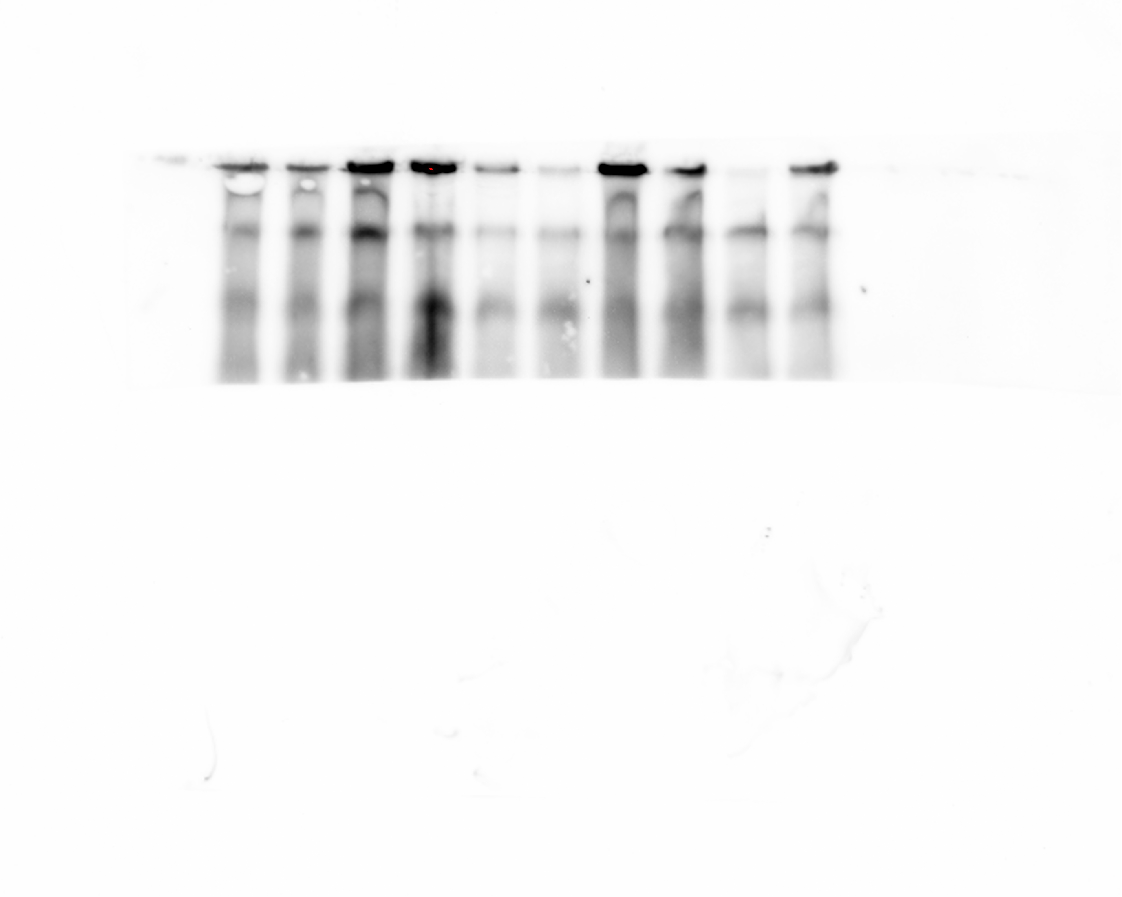

Supplement: Supplementary file 1 [file DataSheet1.ZIP › MMP9 1-2.tif]
